# A Postural Tremor Highly Responsive to Transcranial Cerebello-Cerebral DCS in ARCA3

**DOI:** 10.3389/fneur.2017.00071

**Published:** 2017-03-03

**Authors:** Florian Bodranghien, Nordeyn Oulad Ben Taib, Lionel Van Maldergem, Mario Manto

**Affiliations:** ^1^Unité d’Etude du Mouvement-GRIM, FNRS, ULB-Erasme, Bruxelles, Belgium; ^2^Service de Neurochirurgie, ULB-Erasme, Bruxelles, Belgium; ^3^Service de Neurochirurgie, CHU-StPierre, Bruxelles, Belgium; ^4^Centre de génétique humaine, Université de Franche-Comté, Besançon, France; ^5^Metabolic Unit, Université de Liège, Liège, Belgium; ^6^Service des Neurosciences, UMons, Mons, Belgium

**Keywords:** tremor, cerebellar ataxia, Purkinje neurons, ANO10, transcranial direct current stimulation

## Abstract

**Background and objectives:**

Cerebellar ataxias are disabling disorders that impact the quality of life of patients. In many cases, an effective treatment is missing. Despite the increasing knowledge on the pathogenesis of cerebellar disorders including genetic aspects, there is currently a gap in the therapeutical management of cerebellar deficits. Cerebellar ataxia associated with ANO10 mutation (ARCA3) presents a disabling cerebellar syndrome. The aim of this study is to report a patient with a marked postural tremor responding to transcranial cerebello-cerebral direct current stimulation (tCCDCS).

**Methods:**

We applied tCCDCS using anodal stimulation over the cerebellum with a return electrode on the contralateral motor cortex. We performed a clinical rating, accelerometry studies, and recordings of voluntary movements at baseline, after sham, and after active tCCDCS.

**Results:**

A dramatic response of postural tremor was observed after tCCDCS, with a major drop of the power spectral density to 26.12% of basal values.

**Discussion:**

The postural tremor of cerebellar ataxia associated with ANO10 mutation was highly responsive to tCCDCS in our patient. This case illustrates that tCCDCS is a novel therapeutic option in the treatment of cerebellar deficits and might represent a promising tool to reduce tremor in ARCA3.

## Introduction

Cerebellar ataxias represent a heterogeneous group of disabling disorders (1). With the exception of small subsets of patients suffering from metabolic diseases or vitamin deficiencies, an effective symptomatic treatment is currently missing for the majority of the so-called degenerative ataxias, hence a great need to identify novel therapies in order to fill in this gap in patient care. Current researches in terms of drug discovery have still not provided an effective medication in numerous cerebellar ataxias.

Transcranial direct current stimulation (tDCS) is a non-invasive method, which is currently applied to understand the physiology of cerebellar circuitry and to promote plasticity in cerebellar disorders (2). This safe technique modulates motor and non-motor cerebellar functions, the physiological effects arising mainly from functional changes in the cerebellum itself (3). tDCS causes a polarity-dependent site-specific neuromodulation of brain activity (4). Anodal tDCS induces a depolarization of the neural tissue below the electrode, resulting in a subthreshold membrane potential shift with an increase in the neural firing rate (5). Experimental studies show that anodal epidural DCS of the cerebellum excites the cerebellar cortex, enhances the spinocerebellar evoked potentials associated with peripheral electrical stimulation, and augments cerebellar blood flow both at the levels of cerebellar cortex and cerebellar nuclei (2, 6, 7). At a molecular level, the mechanisms of action include the modulation of ionic gradients in the extracellular space, regulation of channels and pumps as well as modulation of receptors/neurotransmitters (2). This suggests that tDCS could be applied in the management of cerebellar ataxias, especially—but not only—if a dysregulation of ionic gradients is suspected. Recent clinical reports underline that tDCS of the cerebellum administered during a single session or repeatedly could emerge as a novel method in the symptomatic therapy of cerebellar ataxias (8, 9).

Cerebellar ataxia associated with ANO10 mutation (ARCA3, ARCA without peripheral neuropathy) is a recently identified autosomal recessive ataxia (10). The main clinical feature is a cerebellar syndrome. ANO10 (transmembrane protein 16K) is a member of the anoctamin family and codes for a calcium-activated chloride channel involved in neuronal excitation (11, 12). Anoctamins can be compared to channel-like proteins, and the presumed mechanism of ataxia in ARCA3 is a disorder of calcium signaling (10–13). Calcium regulation is a physiological mechanism, which is critical for the Purkinje cell layer in the cerebellar cortex (10, 13).

On the basis of experimental studies in rodents (6) and clinical reports showing an effect of tDCS upon cerebellar ataxias (8, 9), we hypothesized that targeting the cerebello-thalamo-cortical pathway with tDCS might be beneficial to reduce tremor in cerebellar ataxia associated with ANO10 mutation. We speculated that anodal tDCS of the cerebellum would antagonize the impaired activation of Cl^−^ currents (13, 14), taking advantage of a return electrode (cathodal effect) located in front of the contralateral motor cortex to inhibit the activity of this latter.

## Case Description

**Figure 1:**
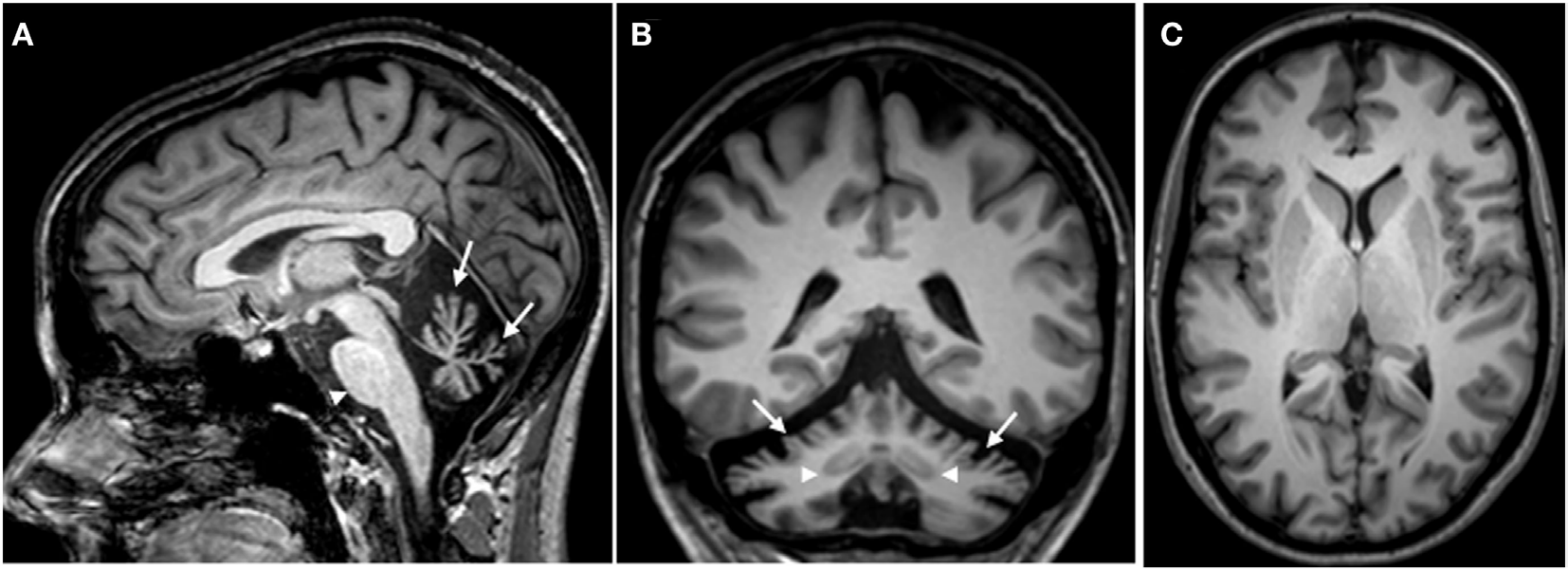
**Brain MRI (T1-weighted images) showing a severe atrophy of the cerebellum in the sagittal plane (A)**. The arrows point to the atrophic vermis, whereas the morphology of the brainstem appears normal (arrowhead). A severe atrophy of the cerebellar cortex is observed in the frontal plane [arrows in panel **(B)**]. Note that the dentate nuclei are clearly identified on both sides (arrowheads). Striatum, thalamus, subcortical white matter, and cerebral cortex appear morphologically normal on both sides [axial image in panel **(C)**].

This 33-year-old right-handed female complained of limbs clumsiness and unsteady gait starting insidiously at the age of 24. Parents were non-consanguineous, there was no family history of cerebellar ataxia, and there was no history of epilepsy. She was taking escitalopram 10 mg/day, baclofen 20 mg/day, and memantine 10 mg/day. She presented a phenotype characterized by a pancerebellar syndrome. She exhibited gaze-evoked nystagmus, hypermetric saccades, scanning speech, dysmetria in four limbs, a steady postural tremor bilaterally in upper limbs, kinetic tremor in four limbs, and ataxia of stance and gait. She showed tendon hyperreflexia in four limbs. Hoffman’s reflex was negative. Plantar reflexes were flexor. Sensory examination (pinprick, light touch, two-point discrimination, position sense, stereognosia) was normal. There was no myoclonus and no dystonia. Genetic study showed a homozygous splice site c.1219-1G>T *ANO10* mutation (chromosome 3p22). Other causes of progressive ataxia were excluded by blood laboratory investigations (15). Brain MRI demonstrated a diffuse cerebellar cortical atrophy sparing cerebellar nuclei (Figure 1).

## Transcranial Cerebello-Cerebral Direct Current Stimulation (tCCDCS) Protocol, Tremor Recording, and Analysis of Dysmetria

The protocol is modified from a previous description (16) and was performed the same day. The patient was comfortably seated in an armchair. Two tri-axial accelerometers (TSD109C1; Biopac; USA) were affixed with tape on the extremity of the right and left index fingers, respectively. The patient was asked to maintain the upper limbs motionless horizontally in front and parallel to the floor. Data were collected on right/left upper limb in three experimental conditions: (1) at baseline, (2) after sham stimulation (20 min of sham stimulation), and (3) after tCCDCS. The rationale for this design baseline–sham–tDCS in cerebellar disorders has been explained elsewhere (7). For sham and anodal stimulation of the cerebellum, the sponge electrode—size: 50 mm × 40 mm—was positioned at the level of the posterior fossa on the right side with the center of the sponge at about 3 cm to the right of the inion, in order to target the right cerebellar hemisphere, given the lateralized cerebellar functions for upper limbs (16). The second sponge electrode—the cathode; same size than anode—was applied over the left motor cortex at the level of the hand representation. Electrodes were soaked with a solution of NaCl 0.9%. The period of stimulation lasted 20 min for both sham stimulation and anodal stimulation. Current delivered was 1.5 mA (portable stimulator CES, Canada). Current was increased gradually from 0 to 1.5 mA over 30 s. For sham stimulation, once the current reached the plateau, it was gradually decreased to 0 over a period of about 1 min, so that the patient was blinded as to whether she was receiving sham stimulation or anodal tDCS. The patient was invited to stay relaxed during the stimulation periods. We applied an “off-line” approach for the recordings of tremor (assessment within 30 min after application of sham or tCCDCS). Three recordings of 30 s (sampling rate: 256 Hz per axis for each accelerometer) were performed in each of the three experimental conditions (basal, post-sham, and post-tCCDCS). We performed the spectral analysis using fast Fourier transform as recommended using Matlab (MathWorks, USA) (17, 18). The 30-s time series were segmented into 10 segments of 3 s. Auto-spectra of 10 sequential 3 s data epochs were averaged to produce smoothed auto-spectra, with mean removal and a Hamming window for each data segment (17). The following spectral parameters were extracted, and means were computed for the three experimental conditions: maximal PSD, peak frequency of power spectra, center frequency (median value of the area below the power spectrum), and frequency dispersion (frequency width of the interval around the center frequency that contains 66% of the total power spectrum) (19, 20). Composite data (square root of the sum of the accelerations squared for all three axes) were processed as reported earlier (20, 21). Data presented in Section “Results” are mean ± SD of the three recordings performed in each experimental condition. We did not assess kinetic tremor because it was much more variable in our patient as compared to the steady postural tremor.

We also assessed the effects of tCCDCS on dysmetria of the wrist using the haptic technology as reported previously (16). Sets of 10 fast flexion pointing movements were recorded for three targets (0.2, 0.3, and 0.4 rad; sampling rate for the position signal: 2,048 Hz). We recorded the surface EMG activities of the flexor carpi radialis (agonist) and extensor carpi radialis (antagonist) muscles (amplification: 1,000, filter settings: 20–500 Hz; Delsys surface electrodes, USA). We averaged each set of 10 movements both for wrist angle data and rectified EMG data. We extracted the mean amplitudes of movements and the onset latencies of antagonist EMG activities (16). Kinematic and EMG data were compared with those obtained previously in a control group using the same experimental conditions (*n* = 8 right-handed healthy subjects, mean age ± SD: 34.8 ± 10.2 years; three women) (22). Values of this control group were used to compute *z* scores in the three experimental conditions as follows:

z=(x−μ)/SD

where *x* is the mean kinematic (or EMG) data for a given target (0.2, 0.3, or 0.4 rad) in the patient, μ is the corresponding mean value in the control group, and SD is the standard deviation of the corresponding parameter in the control group.

At the end of the recording session, we asked the patient whether she could identify the order of stimulation (sham versus anodal). She did not feel any difference between the sham stimulation and the anodal stimulation.

## Results

The tolerance to the procedure was excellent. The patient reported a transient sensation of tingling which was similar during sham and active stimulation. There was no redness of the skin.

Values of the clinical evaluation on the basis of Scale for the Assessment and Rating of Ataxia rating scale at baseline, after the sham procedure, and after the tCCDCS procedure are given in Table 1.

**Table 1:** **Clinical rating of ataxic deficits**.^a^

Item of the scale	Score—basal condition	Score—sham condition	Score—transcranial cerebello-cerebral direct current stimulation condition
Gait	3	3	3
Stance	3	3	2
Sitting	1	1	1
Speech	1	1	1
Finger-chase	Right: 2	Right: 2	Right: 1
	Left: 2	Left: 2	Left: 2
	Mean: 2	Mean: 2	Mean: 1.5
Nose-finger	Right: 2	Right: 2	Right: 1
	Left: 2	Left: 2	Left: 2
	Mean: 2	Mean: 2	Mean: 1.5
Fast alternating movements	Right: 1	Right: 1	Right: 0
	Left: 1	Left: 1	Left: 1
	Mean: 1	Mean: 1	Mean: 0.5
Heel-shin	Right: 2	Right: 2	Right: 2
	Left: 2	Left: 2	Left: 2
	Mean: 2	Mean: 2	Mean: 2

*^a^On the basis of Scale for the Assessment and Rating of Ataxia rating scale*.

**Figure 2:**
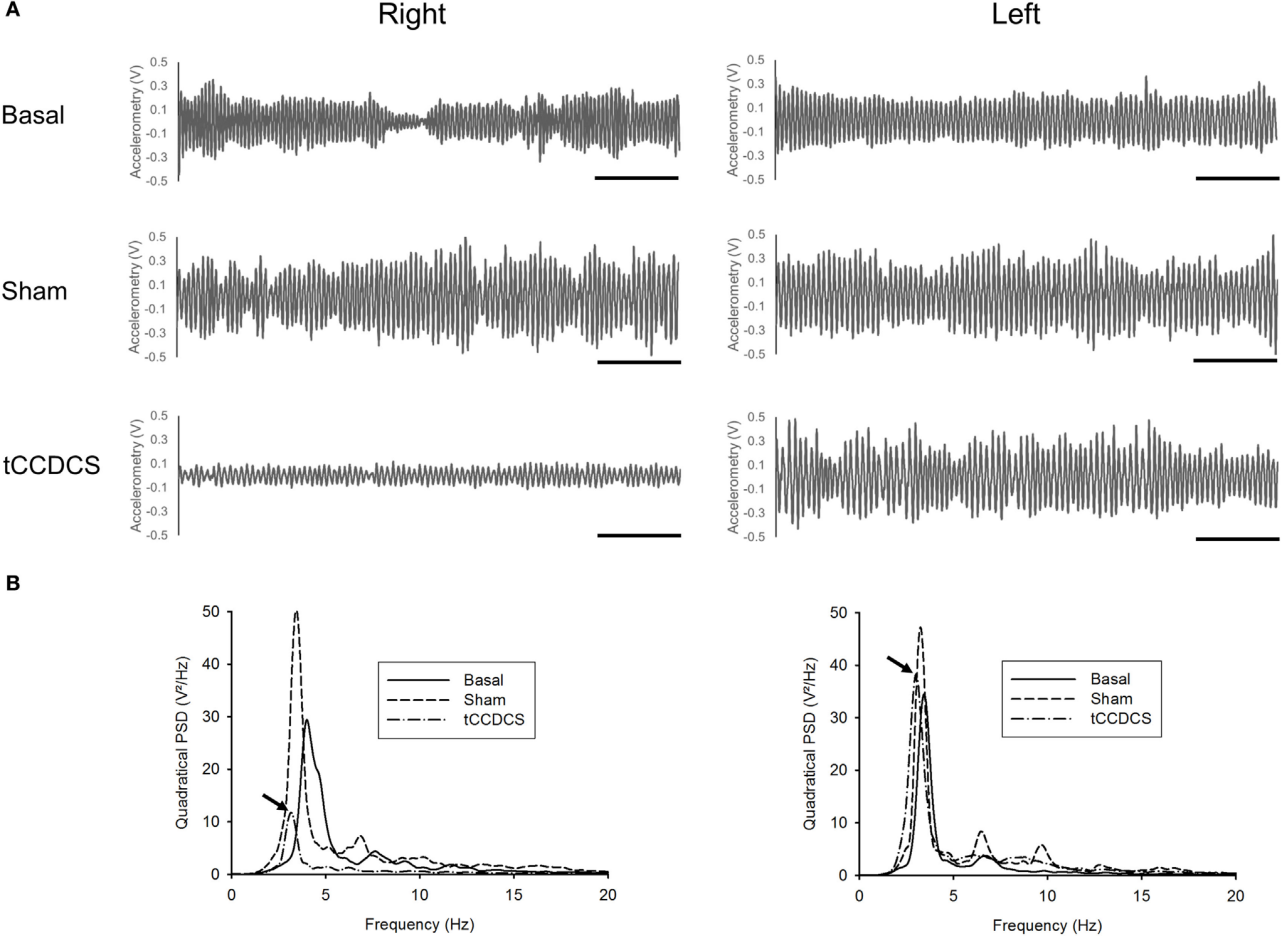
**Effects of transcranial cerebello-cerebral direct current stimulation (tCCDCS) on postural tremor on the right side (left panels) and left side (right panels)**. **(A)** Traces of accelerometry (dominant axis: vertical axis *Y*) at baseline, after administration of sham stimulation, and after application of tCCDCS. Note the strong reduction of tremor oscillations on the right side after tCCDCS. **(B)** Power spectra related to the corresponding tridimensional composite data (*XYZ*). Continuous lines: basal power spectrum, dashed: post-sham, and dot and dashed: post-tCCDCS. Arrows point to the peak power spectral density in the tCCDCS condition. Note the marked drop on the right side after tCCDCS.

On the right upper limb, tCCDCS markedly reduced the postural tremor (see also Supplementary Figure S1 for a time-frequency representation). Figure 2 illustrates a representative recording of the postural tremor along the gravity axis (axis with the most intense oscillations) in the three conditions (basal, post-sham, and post-tCCDCS). A marked reduction in the amplitudes of the oscillations occurred on the right side after active stimulation. The quadratical power spectral density (PSD) changed to 127.43% of baseline values after sham stimulation and dropped markedly to 26.12% after tCCDCS. On the left upper limb, quadratical PSD remained within the 99% confidence interval of basal values (Table 2).

**Table 2:** **Effects of transcranial cerebello-cerebral direct current stimulation (tCCDCS) on spectral parameters of postural tremor**.

Spectral parameter	Right side^a^			Left side^a^		
	Basal	Sham	tCCDCS	Basal	Sham	tCCDCS
Peak power spectral density (V^2^/Hz)	35.07 ± 5.12	44.69 ± 6.22 (1.88)	9.16 ± 2.79^b^ (−5.06)	39.81 ± 4.71	43.05 ± 4.65 (0.69)	40.41 ± 2.82 (0.13)
Peak frequency (Hz)	4.12 ± 0.16	3.48 ± 0.27^b^ (−4.13)	3.33 ± 0.16^b^ (−5.09)	3.32 ± 0.10	2.89 ± 0.56^b^ (−4.41)	3.11 ± 0.11 (−2.15)
Center frequency (Hz)	4.63 ± 0.11	4.09 ± 0.15^b^ (−4.72)	3.87 ± 0.37^b^ (−6.71)	3.63 ± 0.10	3.80 ± 0.02 (1.76)	3.58 ± 0.05 (−0.57)
Frequency dispersion (Hz)	2.26 ± 0.83	2.27 ± 0.88 (0.01)	2.12 ± 0.30 (−0.16)	1.36 ± 0.18	1.90 ± 0.08^b^ (2.94)	1.96 ± 0.07^b^ (3.27)

*^a^Values in brackets correspond to the *Z*-score (as compared to basal values)*.

*^b^Mean values are outside mean ± 2.57 SD of basal values (99% confidence interval)*.

On the right side, hypermetria of fast wrist movements slightly improved after tCCDCS (with a reduction in the delay of the antagonist EMG activity) but values still remained outside the 99% confidence interval for the aimed target located at 0.2 rad (Table 3). On the left side, no change of dysmetria was observed.

**Table 3:** **Effects of transcranial cerebello-cerebral direct current stimulation (tCCDCS) on kinematic and EMG parameters associated with fast goal-directed movements**.

Parameter	Target (rad)	Right side^c^			Left side^c^		
		Basal	Sham	tCCDCS	Basal	Sham	tCCDCS
Mean movement amplitude (rad)^a^	0.2	0.2715 ± 0.0221^d^ (5.67)	0.2711 ± 0.0238^d^ (5.64)	0.2470 ± 0.0154^d^ (3.71)	0.2663 ± 0.0289^d^ (5.25)	0.2669 ± 0.0294^d^ (5.30)	0.2671 ± 0.0292^d^ (5.32)
	0.3	0.3546 ± 0.0245^d^ (3.72)	0.3449 ± 0.0239^d^ (3.05)	0.3328 ± 0.0177 (2.22)	0.3492 ± 0.0302^d^ (3.35)	0.3488 ± 0.0307^d^ (3.32)	0.3490 ± 0.0301^d^ (3.33)
	0.4	0.4612 ± 0.0256^d^ (4.62)	0.4605 ± 0.0254^d^ (4.57)	0.4287 ± 0.0183 (2.20)	0.4514 ± 0.0306^d^ (3.89)	0.4516 ± 0.0299^d^ (3.91)	0.4515 ± 0.0305^d^ (3.90)
Onset latency of antagonist EMG activity (ms)^b^	0.2	112^d^ (9.5)	110^d^ (9.25)	89^d^ (6.63)	105^d^ (8.63)	109^d^ (9.13)	108^d^ (9.0)
	0.3	108^d^ (7.56)	107^d^ (7.44)	85^d^ (5.0)	106^d^ (7.33)	110^d^ (7.78)	103^d^ (7.0)
	0.4	104^d^ (5.36)	102^d^ (5.18)	84^d^ (3.55)	103^d^ (5.27)	102^d^ (5.18)	105^d^ (5.45)

*^a^Control values: target at 0.2 rad: 0.2006 ± 0.01251 rad; target at 0.3 rad: 0.3006 ± 0.01451; target at 0.4 rad: 0.3992 ± 0.01341. Values of the patient are mean ± SD*.

*^b^Control values: target at 0.2 rad: 36 ± 8 ms; target at 0.3 rad: 40 ± 9 ms; target at 0.4 rad: 45 ± 11 ms*.

*^c^Values in brackets correspond to the *Z*-score (as compared to control values)*.

*^d^Mean values are outside mean ± 2.57 SD of control values (99% confidence interval)*.

Subjectively, the patient reported that the right hand was shaking much less after anodal stimulation (“ma main droite tremble beaucoup moins”). She noticed an improvement of manual dexterity on the right side, which lasted about 6 h. She did not report any change on the left side.

## Discussion

Although our findings are novel and open novel door for research in a rare disorder and beyond, there is a need to confirm them in a large group of patients given that our description is based on a single case. Another limitation of the study is the lack of successive recordings during repeated administrations over several weeks in order to characterize the dynamic profile of the response and the impact on daily life. A longitudinal study should be carried out.

Recent reports have highlighted that a single session of anodal DCS of the cerebellum may be beneficial in terms of transient reduction of symptoms in cerebellar disorders (8). The current hypothesis is that anodal DCS of the cerebellum restores the inhibitory effect exerted by Purkinje neurons upon cerebellar nuclei, restoring appropriate patterns of nuclear discharges (2). This disfacilitation of cerebellar nuclei would improve motor control. In our patient, tCCDCS was very active to reduce postural tremor, but the effects on cerebellar dysmetria were less pronounced, although a slight improvement was observed on the right side including in the errors of timing of agonist–antagonist EMG discharges during the execution of fast goal-directed movements.

The central oscillatory network generating limbs tremor includes the primary motor cortex M1 (23). Therefore, it is not surprising that M1 has been considered as a potential target to reduce tremor (24). The fact that M1 is easily accessible to non-invasive stimulation methods renders this potential therapeutic target very attractive to protocols aiming to reduce corticospinal excitability in human tremor disorders, including essential tremor that is known to be associated with an impaired circuitry in the cerebellar cortex (25).

The excitability of the contralateral motor cortex is impaired in both acute and chronic cerebellar lesions (26, 27). In case of hemicerebellar ablation (including removal of cerebellar nuclei), the reduction in the excitability of the motor cortex is antagonized by trains of anodal tDCS applied over the motor cortex (28). We reported earlier that the successive application of anodal stimulation of the cerebellum (cathode over supra-orbital area) and anodal stimulation of the contralateral motor cortex (cathode over supra-orbital area) reduces the postural tremor in spinocerebellar ataxia type 2 (16). Our current setup was different: we aimed to stimulate the cerebellar cortex and to concomitantly inhibit the contralateral motor cortex, *at the same time unbalancing the activities of the two poles of the cerebello-thalamo-cortical pathway*. To explain our observation, we speculate that the *ANO10* mutation resulted in a state of underactivity of the cerebellar cortex, thus disinhibiting the dentato-thalamo-cortical tracts and causing aberrant nuclear discharges, and that tCCDCS (a) restored the inhibitory action of Purkinje neurons upon cerebellar nuclei and (b) reduced the activity of the motor cortex, hence the major reduction of postural tremor. Detailed investigations of the cerebellum–brain inhibition (CBI) and surround motor inhibition before and after tCCDCS should be performed in ARCA3. This could be done during a double-blind, sham-controlled randomized trial (29). The recent work of Benussi et al. has demonstrated a significant improvement in ataxia rating scales, functional tests, and CBI following repeated application of anodal cerebellar tDCS in various forms of cerebellar ataxia (9). Cumulative effects of repeated sessions of stimulation deserve specific studies (9). Moreover, the fact that Cl^−^ currents are involved in apoptosis emphasizes the need to explore whether DCS techniques might exert a neuroprotective effect in ARCA3 (30, 31). Symptomatic improvement of clinical deficits remains a major goal in cerebellar ataxias, but the heterogeneity of these disorders is high, including the mechanisms of neurodegeneration (1).

## Conclusion

We report a major reduction of postural tremor in ARCA3 using tCCDCS. A confirmation in a large group of patients is warranted for establishing tCCDCS as a future standard line of treatment. A collaborative study will be required given the rarity of ARCA3.

## Ethics Approval

Ethics approval was provided by the ULB-Hôpital Erasme Ethics Committee. The patient gave the written informed consent prior to inclusion and following full explanation of the experimental procedures, in accordance with the declaration of Helsinki.

## Author Contributions

FB, NT, and MM: conception/design/execution of the project; LM: genetic analysis; MM: statistical analysis; MM and FB: data analysis, software writing; all the authors: manuscript preparation and corrections of the manuscript.

## Conflict of Interest Statement

The authors declare that the research was conducted in the absence of any commercial or financial relationships that could be construed as a potential conflict of interest. The reviewer GL and handling Editor declared their shared affiliation, and the handling Editor states that the process nevertheless met the standards of a fair and objective review.

## Supplementary Material

Figure S1 **Time–frequency representation of postural tremor at baseline (left panels), after sham stimulation (middle panels), and after transcranial cerebello-cerebral direct current stimulation (tCCDCS) (right panels)**. Power spectrum density for the three axes. Data are shown per frequency and per 1 s time window. Right side: upper panels, left side: lower panels.
