# Supplementary figures and images for: A Postural Tremor Highly Responsive to Transcranial Cerebello-Cerebral DCS in ARCA3
Source: Front Neurol. 2017 Mar 3;8:71. doi: 10.3389/fneur.2017.00071 (PMC5334604; doi:10.3389/fneur.2017.00071)

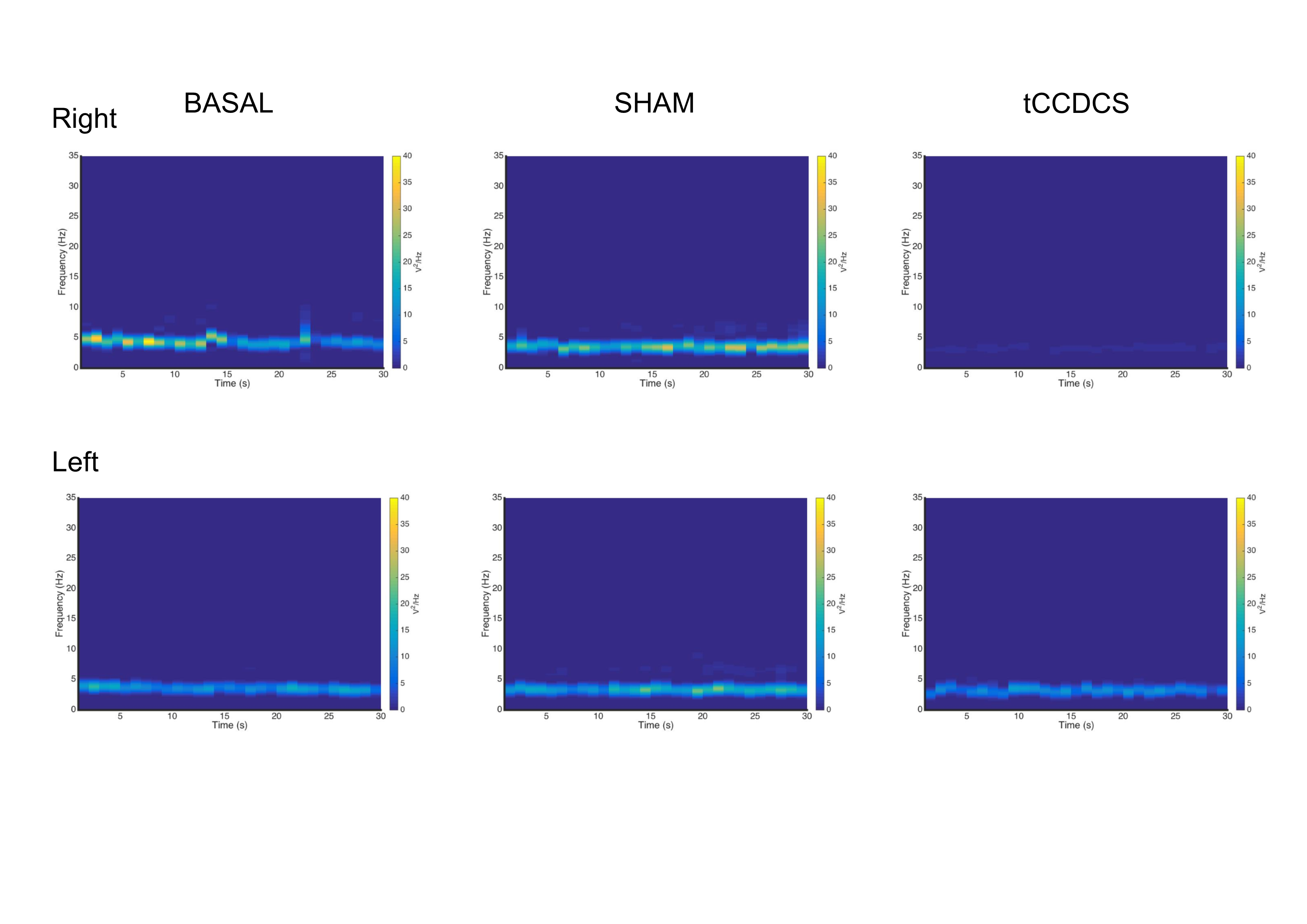

Supplement: Figure S1 — Time–frequency representation of postural tremor at baseline (left panels), after sham stimulation (middle panels), and after transcranial cerebello-cerebral direct current stimulation (tCCDCS) (right panels). Power spectrum density for the three axes. Data are shown per frequency and per 1 s time window. Right side: upper panels, left side: lower panels. [file Image_1.tif]
